# Frameworks for implementation of policies promoting healthy nutrition and physically active lifestyle: systematic review

**DOI:** 10.1186/s12966-021-01242-4

**Published:** 2022-02-12

**Authors:** Karolina Lobczowska, Anna Banik, Piotr Romaniuk, Sarah Forberger, Thomas Kubiak, Biljana Meshkovska, Agnieszka Neumann-Podczaska, Krzysztof Kaczmarek, Marie Scheidmeir, Janine Wendt, Daniel A. Scheller, Katarzyna Wieczorowska-Tobis, Juergen M. Steinacker, Hajo Zeeb, Aleksandra Luszczynska

**Affiliations:** 1 Psychology Department in Wroclaw, SWPS University of Social Sciences and Humanities, Ostrowskiego Street 30b, PL53238 Wroclaw, Poland; 2 Department of Health Policy, School of Health Sciences in Bytom, Medical University of Silesia in Katowice, 18 Piekarska Street, PL41902 Bytom, Poland; 3 Leibniz Institute for Prevention Research and Epidemiology - BIPS, Achter Street 30, D28359, Bremen, Germany; 4 Institute of Psychology, Johannes Gutenberg University Mainz, Binger Street 14-16, D55122 Mainz, Germany; 5 Department of Nutrition, Institute of Basic Medical Sciences, University of Oslo, PO Box 1046, Blindern, 0316 Oslo, Norway; 6 Department of Palliative Medicine, Poznan University of Medical Sciences, Russa Street 55, PL61245 Poznan, Poland; 7 Department of Internal Medicine, Division of Sports and Rehabilitation Medicine, University Hospital Ulm, Leimgrubenweg 14; D89075, Ulm, Germany; 8 Melbourne Centre for Behavior Change, Melbourne School of Psychological Sciences, University of Melbourne, Redmond Barry Building, Parkville Campus, VIC 3010 Melbourne, Australia

**Keywords:** Theory, Framework, Policy, Implementation, Nutrition, Diet, Physical activity, Sedentary behavior, Systematic review

## Abstract

**Background:**

Policy frameworks focusing on policy implementation may vary in terms of their scope, included constructs, relationships between the constructs, and context factors. Although multiple policy implementation frameworks exist, the overarching synthesis characterizing differences between the frameworks is missing. This study investigated frameworks guiding implementation of policies aiming at healthy nutrition, physical activity promotion, and a reduction of sedentary behavior. In particular, we aimed at examining the scope of the frameworks and the content of included constructs (e.g., referring to implementation processes, determinants, or implementation evaluation), the level at which these constructs operate (e.g., the individual level, the organizational/community level), relationships between the constructs, and the inclusion of equity factors.

**Methods:**

A systematic review (the PROSPERO registration no. CRD42019133251) was conducted using 9 databases and 8 stakeholder websites. The content of 38 policy implementation frameworks was coded and analyzed.

**Results:**

Across the frameworks, 47.4% (18 in 38) addressed three aims: description of the process, determinants, and the evaluation of implementation. The majority of frameworks (65.8%; 25 in 38) accounted for constructs from three levels: individual, organizational/community, and the system level. System-level constructs were included less often (76.3%; 29 in 38) than individual-level or organizational/community-level constructs (86.8% [33 in 38 frameworks] and 94.7% [36 in 38 frameworks] respectively). The majority of frameworks (84.2%, 32 in 38) included at least some sections that were solely of descriptive character (a list of unassociated constructs); 50.0% (19 in 38) included sections of prescriptive character (general steps of implementation); 60.5% (23 in 38) accounted for explanatory sections (assuming bi- or uni-directorial associations). The complex system approach was accounted for only in 21.1% (8 in 38) of frameworks. More than half (55.3%; 21 in 38) of frameworks did not account for any equity constructs (e.g., socioeconomic status, culture).

**Conclusions:**

The majority of policy implementation frameworks have two or three aims (combining processes, determinants and/or the evaluation of implementation), include multi-level constructs (although the system-level determinants are less frequently included than those from the individual- or organizational/community-level), combine sections of purely descriptive character with sections accounting for prescriptive and/or explanatory associations, and are likely to include a little or no equity constructs.

**Registration:**

PROSPERO, #CRD42019133251.

**Supplementary Information:**

The online version contains supplementary material available at 10.1186/s12966-021-01242-4.

## Background

As the number of deaths attributable to poor diet and low levels of physical activity (PA) has been increasing over the last two decades [1], the number of public policies aiming at changes in dietary and physical activity behaviors has been growing [2]. For example, the World Cancer Research Fund [2] identified almost 700 national-level healthy diet policies and over 150 national-level PA policies. Policies are actions developed and implemented to directly or indirectly achieve specific goals within a society, for example, better health through better nutrition and PA, or a reduction of sedentary behavior (SB) [3]. Public policies entail a participation of national or regional governments that are involved in developing and implementing policies [3].

Policy implementation may be defined as translating policy goals into actions or integrating a policy within a setting or a system, or the actions aimed at maintaining the use and capacity of a policy [4]. Policy implementation refers to actions through which policies are operationalized within an organization, a community, or a society [5]. More than 60 approaches explaining implementation and dissemination of health-promoting interventions or policies were identified by Tabak et al. [6] whereas Nielsen and Bernjardsson [7] listed 17 frameworks/checklists of barriers and facilitators that influence implementation. Both reviews [6, 7] discussed frameworks without specifying if they refer to implementation of policies aiming at specific health outcomes. In contrast, Flynn et al. [8] identified 7 frameworks that may be used for the evaluation of implementation of policies targeting healthy diet and PA promotion.

Policy implementation frameworks are a subtype of policy frameworks, focusing on ways a policy is put into action [9]. Implementation frameworks are graphical or narrative representations of the key constructs to explain the phenomenon of implementation, and as a minimum they need to include the implementation processes (e.g., stages) or constructs relevant for implementation [4]. Comparisons between policy frameworks may address their specificity (e.g., general health-related actions vs. specific health outcomes the framework addresses), their content or their aims (e.g., explaining implementation processes, identifying implementation determinants, or describing implementation evaluation), the level at which the constructs operate (e.g., individual-, organizational-, or system-levels), relationships between the constructs (e.g., a lack of associations, uni- or bi-directorial associations), and the inclusion of a broader sociodemographic and economic context [9].

Not all implementation determinants or implementation processes can be anticipated during the policy development, therefore constant evaluation of policy implementation is required to adjust implementation and to enable the target groups to actively engage with the policy, and benefit in terms of better health [10]. In line with these observations, Nilsen [11] proposed that frameworks explaining implementation of health-related actions may have one of three types of scopes and include respective scope-related constructs. Process frameworks describe the steps and practical guidance in the planning and execution of implementation endeavors [11]. Determinant frameworks focus on barriers and facilitators that influence implementation processes and their outcomes. Finally, evaluation frameworks define how to assess implementation processes or specify which implementation outcomes should be measured [11]. The approach developed by Nilsen [7, 11] suggests that implementation frameworks belong to either of the three types. However, some frameworks may have a complex scope, for example attempt to explain implementation processes as well as their determinants [5].

In context of obesity-preventing behaviors, implementation of multi-level polices or interventions may have the highest public health potential and result in behavior change [12]. The frameworks guiding policy implementation may differ in terms of the levels accounted for, but even early frameworks explaining implementation of healthy nutrition and PA promotion accounted for individual-level factors and environmental-level factors [13]. According to the evidence-informed policy and practice approach to policies [14] the environmental level may be further divided into the organizational or community level, referring to processes, determinants or implementation evaluation taking place in a target organization or community, and a system level, referring to external lobbying groups, co-existing governmental policies and regulations, administrative structures, etc.

The constructs included in policy implementation frameworks may form different types of relationship. The framework to knowledge approach [15, 16] suggests that any frameworks may be classified into four types, depending on the associations between the constructs. Descriptive frameworks describe the key constructs, including their properties, characteristics, and/or qualities, without assuming any specific relationships between the constructs [15, 16]. Prescriptive frameworks provide a general direction of the actions, explaining them in a series of steps to be taken. Explanatory frameworks include more specific uni- or bi-directional associations between domains (or concepts within the domains) contained within a framework. Predictive frameworks hypothesize directional relationships between all constructs included in the framework [15, 16].

Besides approaches focusing on linear associations between the constructs included in frameworks, the complex system approaches recognize that the constructs may be associated in a non-linear and multi-directional way [17, 18]. Complex system approaches assume that systems are more than the sum of the domains, constructs, and relationships between them [18]. For example, the associations between the constructs may take a form of feedback loops; a change in a feedback loop linking two constructs may result in changes reverberating throughout the system [18].

The main goal of public health policies may be described as promoting better health for everyone [19]. Therefore, an inclusion of equity-related constructs may be yet another dimension allowing to categorize implementation frameworks. When health policies and their implementation are considered, the key equity factors refer to economic status, education, gender, age, ethnicity, geographic isolation, and culture [19, 20]. For example, considering geographic isolation may foster implementation of healthy diet policies that would reach the individuals and communities in remote locations, whereas considering culture may result in including strategies for training cultural competences of the implementers [5].

Although several reviews of implementation frameworks exist [6–8, 11], they have some limitations. First, these reviews focus on various types of actions [6–8, 11], but none of them purposefully investigated policy-related frameworks. Compared to frameworks developed to guide interventions (focused on individual’s behaviors, beliefs, and skills), policy frameworks may have their specificity, for example related to the role of the political context and involved institutions [9]. To the best of our knowledge, reviews of *policy* implementation frameworks, developed or applied to promote healthy diet and PA, are missing. Second, the existing reviews of implementation frameworks compare the frameworks in one preselected area, for example in terms of the aspects of evaluation [8] or implementation determinants [7]. Research on developments in policy frameworks suggested that there are several areas of key differences, referring to the scope of such frameworks, the types of included constructs, relationship between the constructs, and the context accounted for [9]. Comparing policy implementation frameworks in terms of their scope, included constructs, relationships between constructs, and the context may offer a useful guide for researchers and practitioners considering which framework to choose and how their chosen framework compares to other frameworks across these critical areas [9].

This review aims at identifying implementation frameworks for policies promoting healthy diet and physically active lifestyle (defined as promotion of physical activity and reduction of sedentary behavior) and characterizing their scope, the content of the frameworks, relationships between the included constructs, and the equity context factors accounted for. In particular, we analyzed if policy implementation frameworks: (1) aimed at identifying implementation processes, implementation determinants (e.g., barriers and facilitators), or implementation process evaluation; (2) accounted for constructs from individual, organizational/community, and system levels; (3) assumed any associations between constructs (and specific types of associations, i.e., if the frameworks were descriptive, prescriptive, explanatory, predictive or applied complex system approaches); and (4) accounted for equity constructs (economic status, education, age, gender, ethnicity, culture, and geographic isolation).

## Methods

### Search strategy

A systematic search of 9 databases of peer-reviewed journal was performed to identify peer-reviewed publications concerning frameworks for implementation of policies targeting nutrition and PA/SB. Next, to cover grey literature, 8 stakeholder databases were searched, consistently with the approach applied in previous reviews on implementation frameworks [8] and implementation determinants [21]. For the full list of searched databases see Supplementary Table S1, Additional File 2. In addition, manual searches of existing reviews and journals aiming at research on policy implementation was performed (e.g., *Policy Studies*). Documents and articles published between inception of the databases and February 2020 were included. The search was conducted using a combination of four groups of keywords (both for peer-reviewed journals and stakeholder databases) referring to: (1) process, determinants, and evaluation of implementation (e.g., implement*); (2) frameworks (e.g., model*); (3) the type of action (e.g., law OR strateg*); (4) behavior (e.g., diet*) (for the full list of keywords see Additional File 2, Supplementary Table S1). The study was conducted in line with the Preferred Reporting Items for Systematic Reviews and Meta-Analyses (PRISMA) guidelines [22, 23]. The review was preregistered with PROSPERO database (no. CRD42019133251). Besides the preregistered research aims, a question referring to the inclusion of the equity constructs into the policy implementation frameworks was added, consistently with recent research and developments in frameworks for policy and policy implementation [5, 8].

### Inclusion and exclusion criteria

The following inclusion criteria were applied: (1) papers and documents discussing an original policy implementation framework (or its significantly revised versions); (2) papers discussing policy implementation in the context of nutrition, and/or PA and/or SB outcomes; (3) stakeholder documents officially approved by the respective organization; (4) only English-language stakeholder documents and peer-reviewed articles. The exclusion criteria were: (1) documents or papers presenting partial frameworks that include only one concept, variable, or factor; (2) documents or papers presenting frameworks that were developed as applicable solely to specific policies, other than promoting healthy nutrition, and/or PA and/or SB (e.g., safety at work policy implementation frameworks); (3) documents or papers presenting a framework that focuses on other aspects of policy than implementation (e.g., policy development frameworks, policy evaluation frameworks, or policy frameworks that used the term/concept of implementation without specifying what is included in the ‘implementation’); (4) dissertations, protocols, conference materials, and book chapters.

### Data collection and extraction

**Fig. 1:**
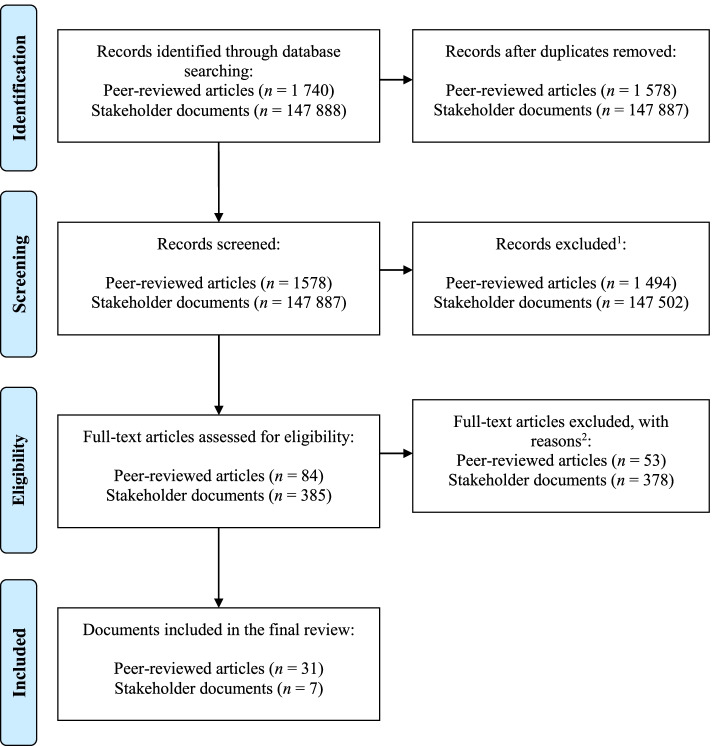
The flow chart: selection processes for peer reviewed articles and stakeholder documents. Note: ^1^ - Records excluded with reasons: document missing any kind of scientific considerations (not a framework, key analyses unrelated to nutrition, physical activity, sedentary behavior; unrelated to policies); ^2^ - Full-text articles excluded, with reasons: not an implementation framework, only mentioning a framework but not the original source of the framework, documents lacking any deeper description or discussion over the frameworks mentioned, documents discussing a general context for policy implementation or strategy, not being put in any structured framework

The initial search resulted in identifying 149,628 potentially relevant documents (see Fig. 1 for the details of data selection). The titles and abstracts of potentially relevant documents were screened. Next, the full-text versions of articles and documents were retrieved and reviewed in terms of their match with inclusion criteria. Overall, we included 31 articles (describing *n* = 31 frameworks) and 7 stakeholder documents (describing *n* = 7 frameworks) meeting all inclusion criteria (see Table 1).

**Table 1:**
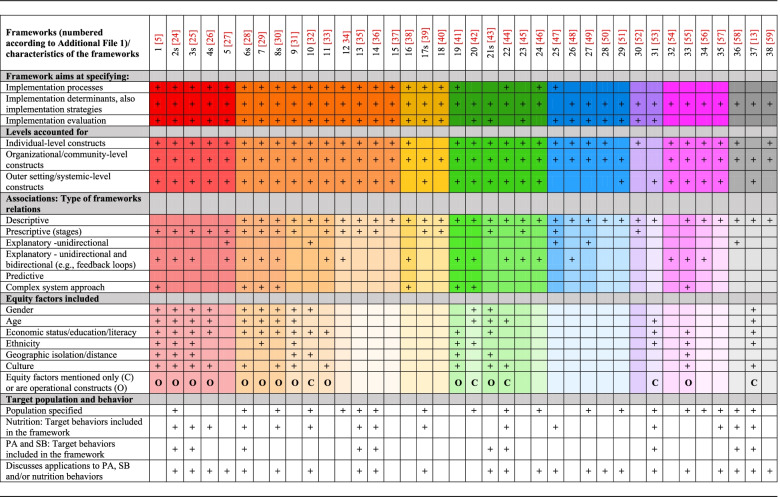
Policy implementation frameworks: aims, levels accounted for, associations between the included constructs, and equity factors

Note: s – the frameworks developed by a stakeholder are indicated with ‘s’ after the framework number; PA - physical activit; SB - sedentary behavior (1) The Context and Implementation of Complex Interventions (CICI) framework­­ [5]; (2s) The DPAS (Global Strategy on Diet, Physical Activity and Health) School Policy Framework) [24]; (3s) Global Strategy on Diet, Physical Activity and Health: A framework to monitor and evaluate implementation DPAS:[25]; (4s) Implementing a salt reduction strategy framework – a practical approach [26]; (5) PRECEDE-PROCEED planning model [27]; (6s) DPAS in the Eastern Mediterranean Region [28]; (7) Practical, Robust Implementation and Sustainability Model (PRISM) [29]; (8s) Stepwise approach: Four Steps to Design Public Food Procurement Initiatives [30]; (9) The pragmatic Ottawa Model of Research Use (OMRU) [31]; (10) Conceptual Model of Evidence-Based Practice Implementation in Public Service Sectors [32]; (11) Conceptual framework of Equity-focused Implementation Research (EquIR) of health programs; [33]; (12) The Nutrition Implementation Framework [34]; (13) Conceptual framework for designing and implementing health promotion programmes in schools [35]; (14) Not specified [36]; (15) Consolidated Framework for Implementation Research,(CFIR) [37]; (16) Normalization Process Model (NPM) [38]; (17s) a framewowk without a specified name [39]; (18) The Quality Implementation Metaframework (QIF) [40]; (19) ) The Advocacy Coalition Framework (ACF) [41]; (20) the multilevel implementation quality framework [42]; (21s) Normalization Process Theory [43]; (22) Steps to health: a European framework to promote physical activity for health [44]; (23) Multilevel Implementation Framework (MIF) [45]; (24) The Behavior Change Ball [46]; (25) Implementation science in nutrition framework (ISN) [47]; (26) Promoting Action onResearch Implementation in Health Services (PARiHS) [48]; (27) Organization theory for determinants of effective implementation of worksite health promotion programs [49]; (28) RE-AIM evaluation model [50]; (29) Framework for a "public health approach"- a global framework for the primary care response to chronic NCDs [51]; (30) Framework for design and evaluation of complex interventions to improve health [52]; (31) The Implementation Framework [53]; (32) the Interactive Systems Framework for Dissemination and Implementation (ISF) [54]; (33) The He Pikinga Waiora (Enhancing Wellbeing) Implementation Framework [55]; (34) Ecological framework for understanding effective implementation [56]; (35) Comprehensive school health framework (CSH) [57]; (36) Conceptual Framework for Organizational Readiness to Implement Nutrition and Physical Activity Programs [58]; (37) The ANalysis Grid for Environments Linked to Obesity ANGELO [13]; (38) Theoretical Domains Framework (TDF) [59]

To address the study objectives the following data were extracted (see Additional File 1, Supplementary Table S1): the target population and behavior (healthy diet, PA, SB), equity factors included in the framework (such as gender, age, ethnicity), the scope of framework and the type of constructs included (processes, determinants, or evaluation of implementation), levels accounted for (individual, organizational/community, system levels or a complex system approach), and the types of associations between the constructs (descriptive, prescriptive, explanatory, or predictive).

All stages of data search, selection, extraction and coding were conducted by at least two researchers. Any disagreements during these stages were resolved by the consensus method (searching for possible rating errors, followed by a discussion and an arbitration by a third researcher [7]).

### Data coding, analysis and synthesis

Data regarding each included policy implementation frameworks were coded according to five categories: (1) the scope of the content of the framework (specifying implementation processes, determinants [and/or strategies] and/or evaluation) [11]; (2) levels of constructs the framework accounts for: the individual-level, the organizational/community-level, and/or the outer setting/system-level [14]; (3) the types of relationships between the framework constructs (was the framework descriptive, predictive, explanatory – unidirectional or bidirectional, predictive or using a complex system approach) [16]; (4) equity factors included in the framework (gender, age, economic status/education/literacy, ethnicity, geographic isolation/distance, culture); (5) frameworks that indicated a direct focus on a particular behaviors (e.g., nutrition included directly into the framework) vs. discussed applications for these behaviors (e.g., nutrition listed as one of potential areas of possible application).

In case a framework included a particular category it was coded as accounting for this criterion (+). Coding was performed following the definitions provided by Nielsen [11] (aims of the framework; see Table 1), Bowen and Zwi [14] (levels that the framework accounts for), Rycroft-Malone and Bucknall [16] (types of relationships), and the Organization for Economic Co-operation and Development as well as Bleich et al. proposals [19, 20] for key equity factors in health policies. Additional File 2 (Supplementary Table S2) provides criteria applied in coding of the extracted data.

The variables for which data were sought were defined as follows:

- *Policies* are decisions, plans and actions developed and implemented to directly or indirectly achieve specific goals within a society, for example, better health through better nutrition and PA, or a reduction of SB [3]. Policies involve a participation of national or regional governments that are involved in developing and implementing policies [3].
- *Implementation* is defined as the process of putting to use or integrating a policy within a setting or a system, or the process of maintaining the use and capacity of a policy [4]. *Public policy implementation* can be considered as a process of carrying out a government decision [60], and reflects a complex change process where government decisions are transformed into programs, procedures, regulations, or practices aimed at social betterment [61].
- *Framework* is defined as “a graphical or narrative representation of the key factors, concepts, or variables to explain the phenomenon of implementation” [4]. Specifically, implementation frameworks should include either implementation steps, or implementation determinants, or strategies [4]. Frameworks may specify the relationships between the included constructs [16]. As the framework development progresses through an integration of new evidence, the constructs may evolve from relatively broad or vague to more specific and well-defined [62].

## Results

Overall, *N* = 38 frameworks were identified. The characteristics of the frameworks, including their aims and respective constructs, associations between constructs, equity factors included, and the target behavior are presented in Table 1.

### The scope and the content of policy implementation frameworks

**Fig. 2:**
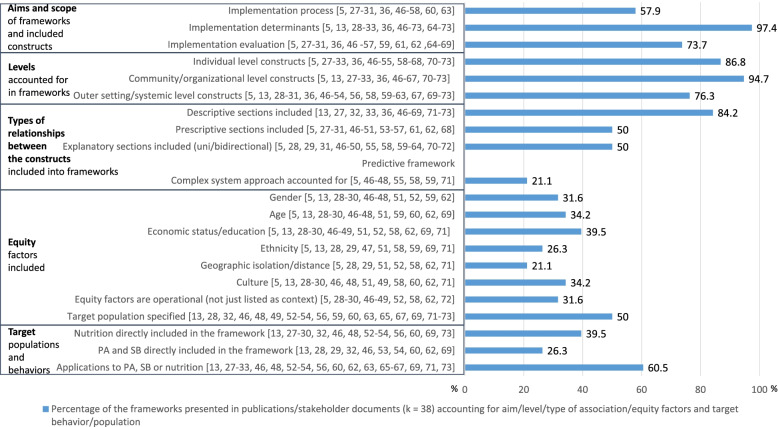
Summary of policy implementation frameworks’ focus. Percentage of policies accounting for aims/focus of the frameworks, levels accounted for, types of frameworks in terms of relationships between the constructs, equity factors, target populations, and target behaviors

Except of one [47], all frameworks (97.4%; 37 in 38 frameworks) included implementation determinants. The majority accounted for evaluation of implementation (73.7%; 28 in 38 frameworks), whereas implementation processes were addressed the least frequently (57.9%; 22 in 38 frameworks; see Fig. 2).

Overall, 7 in 38 (18.4%) frameworks accounted for one type of content-related constructs only (either process only, or determinants only, or evaluation only), whereas 18 in 38 (47.4%) frameworks included a mix of all three types of constructs (the process, determinants, and the evaluation). Nine (23.7%) in 38 frameworks addressed both implementation determinants and the evaluation of implementation. The smallest number of frameworks aimed at describing two remaining scopes, namely implementation processes and implementation determinants (7.9%, 3 in 38), or combined aims referring to implementation processes and implementation evaluation (2.6%, 1 in 38).

### Individual, community, and system levels in policy implementation frameworks

The majority of policy implementation frameworks (65.8%, 25 in 38) included constructs from all three analyzed levels (individual, organizational/community, and system), whereas only 7.9% (3 in 38) accounted for one level only. Organizational/community-level variables were included in almost all frameworks (94.7%; 36 in 38). Individual-level constructs were included in the majority of frameworks (86.8%; 33 in 38). The system-level constructs were accounted for in majority of frameworks as well (76.3%; 29 in 38), albeit the least frequently (Fig. 2).

### Relationships between the constructs included in policy implementation frameworks

Overall, 84.2% of frameworks (32 in 38) included at least some sections which were of solely descriptive character (a list of constructs that were not associated in any specific manner). A half of the frameworks (50.0%, 19 in 38) included sections that were of prescriptive character (e.g., stages). The majority of frameworks (60.5%, 23 in 38) included explanatory sections assuming uni-directorial and/or bi-directorial associations between included constructs. Bi-directorial associations were indicated in 50.0% (19 in 38) of frameworks. Elements of the complex system approach were included in 21.1% (8 in 38) frameworks only (Fig. 2).

Across the frameworks, 18.4% (7 in 38) were of descriptive character solely, one framework (2.6%) was of prescriptive only, and one (2.6%) was solely of explanatory character. None of the frameworks was of predictive character, that is assuming that all constructs are linked in a specific (uni- or bi-directional) manner. The remaining 29 frameworks (76.3%) combined aspects typical of descriptive, predictive, or explanatory frameworks. In particular, 18.4% (7 in 38) of frameworks included sections which were of solely descriptive character combined with sections presenting prescriptive associations, whereas 28.9% (11 in 38) of frameworks included descriptive sections combined with sections assuming explanatory associations (uni- or bi-directional). Furthermore, 10.5% (4 in 38) frameworks included prescriptive and explanatory sections combined. Finally, 18.4% (7 in 38) of frameworks included sections combining descriptive, prescriptive, and explanatory (uni- or bi-directional) associations.

### Equity factors included in policy implementation framework

Overall, the findings indicate that across the included frameworks 55.3% (21 in 38) did not account for any of the equity factors included in this study, whereas 10.5% (4 in 38) accounted for all six equity factors investigated in the present review (Fig. 2).

Thirteen (34.2% of 38) frameworks included from 2 to 5 equity factors. Economic factors were most frequently included in the frameworks (39.5%, 15 in 38), followed by age and culture-related factors (both accounted for in 34.2%, 13 in 38 frameworks), and gender (31.6%, 12 in 38 frameworks). The least frequently included equity factors were ethnicity (26.3%, 10 in 38 frameworks) and geographic isolation/infrastructure (21.1%, 8 in 38 frameworks).

### Target behaviors included in policy implementation frameworks

Regarding the target behavior, 60.5% (23 in 38) of policy frameworks included nutrition, PA, and SB-related behaviors into the framework directly, or indicated that the framework may be used for implementation of healthy nutrition, PA and/or SB policies. The remaining 39.5% (15 in 38) of frameworks were generic in terms of the target behavior for the policy implementation, which means that they did not indicate that they were developed to guide implementation of policies targeting change of specific health behaviors (Fig. 2).

In particular, 39.5% (15 in 38) of all frameworks directly referred to implementation of healthy nutrition policies, whereas 26.3% (10 in 38) referred to implementation of physical activity or sedentary behavior-related policies. Among these, 36.0% (9 in 25) frameworks addressed both nutrition and PA/SB, 24.0% (6 in 25) addressed nutrition only and 4.0% (1 in 25) addressed PA/SB only.

Across all policy implementation frameworks, 50.0% (19 in 38) specified a target population. The remaining 50.0% (19 in 38) frameworks did not focus on any specific population.

### The frameworks with a complex scope versus frameworks focused on a specific scope

As presented in Table 1, 39.5% (15 in 38) frameworks accounted for all three investigated scopes (processes, determinants, evaluation of implementation) and the three levels (individual, community and outer setting/systemic level). Among them, 13.2% (5 in 38) provided some clarification on the associations between all constructs included in the framework [5, 24–27], whereas 26.3% (10 in 38) included some areas of the framework which were of the descriptive character. Among these 5 frameworks addressing all scopes, levels, and avoiding purely descriptive sections, only 3 included all analyzed equity factors (gender, age, economic status, ethnicity, geographic isolation, and culture) [5, 24, 25]. There were 7 (18.4% of 38) frameworks that addressed only one aim, and thus had a narrowed-down and specific scope [13, 55–59]. All of these 7 frameworks [13, 55–59] focused on listing implementation determinants. Four of these frameworks addressed 3 levels (individual, organizational/community, and system-related) and 6 included some areas of descriptive character (i.e., without specifying the relationship between the included constructs).

## Discussion

This review identified 38 implementation frameworks developed for (or applied in the context of) healthy nutrition, PA promotion, and SB reduction policies. The findings indicate that almost half (47.4%, 18 in 38) of the frameworks had a complex scope, combining aims and constructs referring to processes, determinants, and evaluation or implementation, with additional 13 frameworks (34.2% of *n* = 38) combining two of these aims. Furthermore, the majority of frameworks (65.8%, 25 in 38) accounted for constructs from all three levels: individual, organizational/community, and the system levels. Regarding the relationships between the constructs, the majority of policy implementation frameworks (84.2%, 32 in 38) included at least some sections that were of solely descriptive character (a list of constructs which were not associated in any specific manner). Slightly more than half (55.3%, 21 in 38) of frameworks did not account for any of the equity-related constructs. Finally, we have found that only 3 (7.9% of 38) frameworks [5, 24, 25] accounted for all 3 investigated scopes (processes, determinants, evaluation of implementation), the 3 levels (individual, community/organizational, and system levels), specified relationship between all included constructs, and addressed all analyzed equity factors.

As suggested in Nilsen’s [11] typology, implementation frameworks may be divided into three distinct groups, depending on their scope: (1) addressing implementation process, (2) implementation determinants, or (3) evaluation of implementation. Our findings indicated that the majority of policy implementation frameworks included more aims than one. Thus, the results are in contrast to the assumption made by Nilsen [7, 11] suggesting that implementation frameworks belong to either of the three types.

The identified frameworks were most likely to include policy implementation determinants and the least likely to describe policy implementation processes. This is in contrast to many definitions, highlighting processual aspects of implementation, e.g., defining implementation as a *process* of putting to use or integrating new practices within a setting [63, 64]. It should be noted that several frameworks that focus mostly on determinants (e.g., the Consolidated Framework of Implementation Research; CFIR [37]) highlight that at least some implementation determinants are process-specific, namely they may be particularly relevant during some implementation processes. Our study shows that frameworks including aspects of implementation processes were also more likely to include community and system-level constructs (besides individual-level-constructs) as well as equity constructs. The inclusion of multi-level constructs and equity factors may result from attempts to explain complexity of the implementation process. For example, such process may account for strategies of engaging key stakeholders, adapting policies to the context, prioritizing implementation goals, monitoring the process of implementation among all involved stakeholders, implementers, and the target population [65]. If such processes strategies are included, the framework is likely to include constructs typical of organization/community or system level, and address the diversity of the target population.

The majority of policy implementation frameworks (65.8%, 25 in 38) used a multi-level approach, accounting for individual, organizational/community, and outer setting/systemic level constructs. This is in line with accumulating evidence, pointing towards the highest public health potential of multi-level actions (i.e., policies or interventions) targeting obesity reduction [12, 13]. On the other hand, the system-level determinants were included less frequently than determinants from the individual and organizational/community levels. Furthermore, even if the system-level constructs were addressed, they were described in a relatively general manner (e.g., accounting for ‘external policies’) [37]. Recent framework-guided research on implementation suggested that to increase the usability of policy implementation frameworks in research and practice, the frameworks should include a higher number of specific system-level constructs such as external funding agent priorities, resource source, resource continuity, and strategic policy alignment [66].

As suggested by the framework to knowledge approach [15, 16], theoretical approaches may evolve in a way that descriptive frameworks represent an early stage of a model development, which progresses to more specific (explanatory) and precisely defining all potential relationships (predictive). Our findings indicated that a majority of policy implementation frameworks included at least some descriptive areas, thus they require further theoretical developments. In line with a framework-based research it may be assumed that an inclusion of more specific links between the sections or the constructs would benefit the use of the framework in research and practice [58].

Elements of the complex system approach [17, 18] were rarely integrated into the policy implementation frameworks. Across the last decade, researchers and practitioners have been advocating for the use of the complex system approach to explain obesity and obesity related behaviors (such as nutrition, PA and SB) [67, 68]. The complex system approach was mostly used to map the determinants of obesity or obesity-related behaviors [68, 69]. Future theoretical developments may benefit from the use of system mapping approach [68] and propose complex system-based policy implementation frameworks.

Last but not least, we found that half of the frameworks did not account for any of the equity constructs analyzed in this review. Previous research aiming at adjusting a more generic implementation framework to a specific context (e.g., care transition from being hospitalized to ambulatory care) showed that tailoring the frameworks results in an inclusion of equity factors, such as age, gender or ethnicity [70, 71]. This is in line with the findings of the present study showing that implementation frameworks which were developed specifically for (or addressing) healthy nutrition, PA or SB policy implementation were more likely to account for the equity factors, compared to the frameworks that were developed as more generic.

While this study has several strengths, many limitations are present. This review did not account for policy implementation frameworks that were developed and mostly used in other contexts than changing nutrition, PA, and SB [9, 72], therefore any conclusions are limited to the frameworks that were already applied in research on these behaviors. We did not include books and book chapters into the systematic review, whereas several frameworks that account for policy implementation were originally presented in these types of sources. One of key criteria for comparisons between policy frameworks may refer to their inclusion of events: (1) anticipated, such as elections that produce limited change or introduce new actors with different ideas, or (2) unanticipated, such as social or natural crises (e.g., the COVID-19 pandemic), or major and technological changes [9, 73]. Such criteria are particularly relevant if sustainability of the policy implementation is considered. The criterion referring to an inclusion of the event was not applied in this study. Future research may need to account for this criterion, but also compare policy implementation frameworks using such criteria as inclusion of characteristics of actors making choices or networks and subsystems of ‘pressure participants’ [9]. Although a large number of original stakeholder documents were retrieved during the search, the majority (147,880 in 147,887) were subsequently excluded during the screening process. It is possible that due to a large number of screened documents some of stakeholder frameworks were not identified. As the number of the policy implementation frameworks increases over time, the findings of this review should be updated in upcoming years in order to integrate newer approaches.

## Conclusions

This study provides an overarching synthesis of frameworks guiding implementation of healthy nutrition and PA/SB policies, summarizing their scope, the content of the included constructs, the level at which the constructs operate, relationships between the constructs, and the inclusion of equity factors. The majority of frameworks have a complex scope (combining process, determinants and/or evaluation of implementation), include multi-level constructs (although system level determinants are less frequently included than those at individual or organizational/community level), combine sections of purely descriptive character with sections accounting for prescriptive and/or explanatory associations, and are likely to include a little or no equity constructs.

By summarizing the characteristics of policy implementation frameworks this review may inform directions for future theoretical developments. In particular, existing frameworks could benefit from integrating equity factors and the complex-system approach thinking. When faced with a myriad of policy implementation frameworks, policy makers, researchers, and policy implementation actors may seek guidance on how to select an optimal framework. The findings of this review may facilitate the process of selecting the framework that represents the best match for their needs and aims. It may also help them to put their chosen framework into the context of other existing frameworks, differing in such aspects as the inclusion of equity factors, systemic-level constructs, or accounting for implementation evaluation.

## Supplementary Information

**Additional file 1: **TableS1 Details of data extraction.

**Additional file 2:** Table S1: Full list of 4 groups of keywords applied in the searching strategy and databases searched. Table S2: Additional coding principles for included policy implementation model/frameworks and definitions of key variables. The list of included peer-reviewed articles and stakeholder documents.

## Data Availability

All data analysed during this study are either secondary (retrieved from original studies included in the review) or included in this published article (and its supplementary information files).

## Abbreviations

ACF: Advocacy Coalition Framework
ANGELO: ANalysis Grid for Environments Linked to Obesity
CFIR: Consolidated Framework for Implementation Research
CICI: Context and Implementation of Complex Interventions framework
CSH: Comprehensive School Health framework
DPAS: Global Strategy on Diet, Physical Activity and Health
EquIR: Conceptual framework of Equity-focused Implementation Research of health programs policies and systems
ISN: Integrated Framework for Implementation Science in Nutrition
MIF: Advocacy Coalition Framework
NPM: Normalization Process Model
OMRU: Ottawa Model of Research Use
PA: physical activity
PARiHS: Promoting Action on Research Implementation in Health Services
PRISM: Practical, Robust Implementation and Sustainability Model
QIF: Quality Implementation Metaframework
RE-AIM: Reach, Efficacy, Adoption, Implementation, Maintenance model
SB: sedentary behavior
TDF: Theoretical Domains Framework
