# Supplementary material for: Frameworks for implementation of policies promoting healthy nutrition and physically active lifestyle: systematic review
Source: Int J Behav Nutr Phys Act. 2022 Feb 12;19:16. doi: 10.1186/s12966-021-01242-4 (PMC8841124; doi:10.1186/s12966-021-01242-4)
Supplement: Supplementary file 2 — Additional file 2: Table S1: Full list of 4 groups of keywords applied in the searching strategy and databases searched. Table S2: Additional coding principles for included policy implementation model/frameworks and definitions of key variables. The list of included peer-reviewed articles and stakeholder documents. [file 12966_2021_1242_MOESM2_ESM.docx]

**Frameworks for implementation of policies promoting healthy nutrition and physically active lifestyle: systematic review**

**Additional file 2**

Table S1 Full list of 4 groups of keywords applied in the searching strategy and databases searched

I. List of 4 groups of keywords:

(1) process, determinants, and evaluation of implementation: implement* OR “process evaluation” OR “policy evaluation” OR “policy assess*” OR “policy measur*” OR “policy outcom*” OR “policy acceptab*” OR “policy adopt*” OR “policy appropriat*” OR “policy cost*” OR “policy feasibil*” OR “policy fidelity” OR “policy penetrat*” OR “policy sustain*” of “policy barrier*” OR “policy facilitat*” OR “policy determinant*” OR OR “policy influenc*” OR “policy enhanc*” OR “policy improv*” OR “policy support*” OR OR “policy transposition” OR “policy enforce*” OR “policy compliance” OR “policy complian*”;

(2) frameworks: theor* OR framework* OR model* OR approach*;

(3) the type of action: polic* OR program* OR strateg* OR practic* OR government* OR law OR regulation* OR “soft law” OR “action plan” OR “action program*” OR “national strategy*”;

(4) the target behavior: “physical activity” OR active OR exercise OR sport OR fitness OR sedentary OR diet OR nutrition OR fat OR sugar* OR sweetened OR salt OR sodium OR snack OR fruit OR vegetable OR fiber OR fibre OR soda OR meal OR food OR “energy intake” OR calorie* OR obes* OR eat*

IIa. List of databases searched to retrieve peer-reviewed publications: PsycINFO, PsycARTICLES, Health Source: Nursing/Academic Edition, MEDLINE, Academic Search Ultimate, AGRICOLA, the Cochrane Database of Systematic Reviews (CDSR), the Database of Abstracts of Reviews of Effects (DARE), Scopus.

IIb. List of databases searched to retrieve stakeholders documents: The National Institute for Health and Care Excellence (NICE; United Kingdom) or the European Commission; World Health Organization, Regional Office for Europe (WHO); Institute of Medicine (IOM; USA), Australian Department of Health (ADH), National Health and Medical Research Council (NHMRC; Australia), Organization for Economic Co-operation and Development (OECD), and Food and Agriculture Organization of the United Nations (FAO)

Note: The keywords were selected based on previous reviews addressing similar issues [1-4]. Applied limiters in published reviews: peer-reviewed publications of reviews applying systematic search of original research (including systematic, scoping, realist reviews, meta-analyses), academic journals, English language only. Applied limiters in stakeholders’ databases: documents approved/published by the respective organization, English language only

References included in the Table S1:

1. Horodyska K, Luszczynska A, Hayes CB, et al. Implementation conditions for diet and physical activity interventions and policies: an umbrella review. BMC Public Health. 2015;15(1):1250. doi:10.1186/s12889-015-2585-5
2. Moullin JC, Sabater-Hernández D, Fernandez-Llimos F, Benrimoj SI. A systematic review of implementation frameworks of innovations in healthcare and resulting generic implementation framework. Health Res Policy Syst. 2015;13:16. doi:10.1186/s12961-015-0005-z
3. Proctor E, Silmere H, Raghavan R, et al. Outcomes for implementation research: conceptual distinctions, measurement challenges, and research agenda. Adm Policy Ment Health. 2011;38(2):65-76. doi:10.1007/s10488-010-0319-7
4. Wolfenden L, Jones J, Williams CM, et al. Strategies to improve the implementation of healthy eating, physical activity and obesity prevention policies, practices or programmes within childcare services. Cochrane Database Syst Rev. 2016;10:CD011779. doi:10.1002/14651858.CD011779.pub

Table S2 Additional coding principles for included policy implementation model/frameworks and definitions of key variables

**Coding principles**

Each framework was coded (see Table 1) regarding the target population and behavior (PA, SB and/or nutrition), equity factors included, the type of framework (specifying implementation processes, implementation determinants or strategies, implementation evaluation, implementation system approach) [1,2], level the framework accounted for (individual-level constructs, community/organizational-level constructs, outer setting/systemic-level constructs, or complex system approach) [3], and the type of the framework (descriptive, prescriptive, explanatory- unidirectional, explanatory bidirectional and unidirectional, predictive) [4]. Coding was performed following the definitions provided by Nielsen [1], Bowen & Zwi [3], Rycroft-Malone & Bucknall [5].

Every time a particular behavior was introduced directly in a model/framework (nutrition behavior, PA, SB), it was coded as targeting the particular type of behavior. However, in cases when a model/framework did not refer to a particular behavior directly, but discussed applications for these behaviors it was coded as discussing applications to PA, SB and/or nutrition behaviors only.

If the framework included equity factors as the main domain in the framework or was directly included in the model it was coded as operational key construct (O). However, in cases when equity factors were only mentioned in the model/framework as important factor that should be accounted for and that should be considered as influencing other key factors included in the model it was coded as general context (C).

According to the levels accounted for in the model/framework, a particular level was coded only if it was listed among the key factors explaining the implementation, however, if a level was mentioned only as additional contextual factor that should accounted for it was not coded.

**Definitions**

*Policies* are decisions, plans and actions developed and implemented to directly or indirectly achieve specific goals within a society, for example, better health through better nutrition and PA, or a reduction of SB [5]. Policies involve a participation of national or regional governments that are involved in co-creating and implementing policies. In contrast, interventions are actions aimed at similar health-related goals but not yet endorsed or implemented by regional or national governments [5].

*Implementation* is defined as the process of putting to use or integrating a policy within a setting or a system, or the process of maintaining the use and capacity of a policy [6].

*Policy implementation* can be considered as process of carrying out a government decision [7], and reflects a complex change process where government decisions are transformed into programs, procedures, regulations, or practices aimed at social betterment [8].

*Framework is* defined as “a graphical or narrative representation of the key factors, concepts, or variables to explain the phenomenon of implementation” [6]. Specifically, implementation frameworks should include either implementation steps, implementation determinants, or strategies [6]. Frameworks may specify the relationships between the included constructs [9]. As the framework development progresses through an integration of new evidence, the constructs may evolve from relatively broad or vague to more specific and well-defined [10].

References included in the Table S2:

1. Nilsen P. Making sense of implementation theories, models and frameworks. Implement Sci. 2015;10(1):53. https://doi.org/10.1186/s13012-015-0242-0
2. Rutter H, Savona N, Glonti K, et al. The need for a complex systems model of evidence for public health. The Lancet. 2017;390(10112):2602-2604. https://doi.org/10.1016/S0140-6736(17)31267-9
3. Bowen S, Zwi AB. Pathways to “Evidence-Informed” Policy and Practice: A Framework for Action. PLOS Med. 2005;2(7):e166. https://doi.org/10.1371/journal.pmed.0020166
4. Rubenstein-Montano B, Liebowitz J, Buchwalter J, McCaw D, Newman B, Rebeck K. A systems thinking framework for knowledge management. Decis Support Syst. 2001;31(1):5-16. https://doi.org/10.1016/S0167-9236(00)00116-0
5. Lakerveld J, Woods C, Hebestreit A, et al. Advancing the evidence base for public policies impacting on dietary behaviour, physical activity and sedentary behaviour in Europe: The Policy Evaluation Network promoting a multidisciplinary approach. Food Policy. 2020;96:101873. https://doi.org/10.1016/j.foodpol.2020.101873
6. Moullin JC, Sabater-Hernández D, Fernandez-Llimos F, Benrimoj SI. A systematic review of implementation frameworks of innovations in healthcare and resulting generic implementation framework. Health Res Policy Syst. 2015;13:16. https://doi.org/10.1186/s12961-015-0005-z
7. Berman P. The Study of Macro and Micro Implementation of Social Policy. Published online January 1, 1978. Accessed March 14, 2021. https://www.rand.org/pubs/papers/P6071.html
8. DeGroff A, Cargo M. Policy implementation: Implications for evaluation. New Dir Eval. 2009;2009(124):47-60. https://doi.org/10.1002/ev.313
9. Rycroft-Malone J, Bucknall T. Using theory and frameworks to facilitate the implementation of evidence into practice. Worldviews Evid Based Nurs. 2010;7(2):57-58. https://doi.org/10.1111/j.1741-6787.2010.00194.x
10. Dubois A, Gadde L-E. “Systematic combining”—A decade later. J Bus Res. 2014;67(6):1277-1284. https://doi.org/10.1016/j.jbusres.2013.03.036

**Full list of references of peer-reviewed articles and stakeholder documents presenting frameworks included in the systematic review:**

1. Pfadenhauer LM. Making sense of complexity in context and implementation: the Context and Implementation of Complex Interventions (CICI) framework. Published online 2017:17.
2. World Health Organization. School policy framework: implementation of the WHO global strategy on diet, physical activity and health, ‎2008. https://www.who.int/dietphysicalactivity/SPF-en-2008.pdf?ua=1. Accessed February 8, 2020
3. World Health Organization. Global Strategy on Diet, Physical Activity and Health: a framework to monitor and evaluate implementation. World Health Organization, 2006. https://www.who.int/dietphysicalactivity/Indicators%20English.pdf. Accessed February 8, 2020
4. World Health Organization. Creating and enabling environment for population-based salt reduction strategies, 2010. World Health Organization. https://apps.who.int/iris/bitstream/handle/10665/44474/9789241500777_eng.pdf?sequence=1&isAllowed=y. Accessed February 8, 2020
5. Green L, Kreuter M. Health Program Planning: An Educational And Ecological Approach. J Name. Published online January 1, 2005
6. World Health Organization. Framework for the implementation of the Global Strategy on Diet, Physical Activity and Health in the Eastern Mediterranean Region, 2010). https://apps.who.int/iris/bitstream/handle/10665/116671/emropub_2010_1273.pdf?sequence=1&isAllowed=y. Accessed February 10, 2020
7. Feldstein AC, Glasgow RE. A practical, robust implementation and sustainability model (PRISM) for integrating research findings into practice. Jt Comm J Qual Patient Saf. 2008;34(4):228-243. https://doi.org/10.1016/s1553-7250(08)34030-6
8. Food and Agriculture Organization of United Nations. Strengthening sector policies for better food security and nutrition results, 2019. Education. http://www.fao.org/3/ca7149en/ca7149en.pdf . Accessed February 10, 2020
9. Eslava-Schmalbach J, Garzón-Orjuela N, Elias V, Reveiz L, Tran N, Langlois EV. Conceptual framework of equity-focused implementation research for health programs (EquIR). Int J Equity Health. 2019;18(1):80. https://doi.org/10.1186/s12939-019-0984-4
10. Menon P, Covic NM, Harrigan PB, et al. Strengthening implementation and utilization of nutrition interventions through research: a framework and research agenda. Ann N Y Acad Sci. 2014;1332:39-59. https://doi.org/10.1111/nyas.12447
11. Graham ID, Logan J. Innovations in knowledge transfer and continuity of care. Can J Nurs Res Rev Can Rech En Sci Infirm. 2004;36(2):89-103
12. Aarons GA, Hurlburt M, Horwitz SM. Advancing a conceptual model of evidence-based practice implementation in public service sectors. Adm Policy Ment Health. 2011;38(1):4-23. https://doi.org/10.1007/s10488-010-0327-7
13. Pearson M, Chilton R, Wyatt K, et al. Implementing health promotion programmes in schools: a realist systematic review of research and experience in the United Kingdom. Implement Sci IS. 2015;10:149. https://doi.org/10.1186/s13012-015-0338-6
14. Wierenga D, Engbers LH, van Empelen P, Hildebrandt VH, van Mechelen W. The design of a real-time formative evaluation of the implementation process of lifestyle interventions at two worksites using a 7-step strategy (BRAVO@Work). BMC Public Health. 2012;12:619. https://doi.org/10.1186/1471-2458-12-619
15. Damschroder LJ, Aron DC, Keith RE, Kirsh SR, Alexander JA, Lowery JC. Fostering implementation of health services research findings into practice: a consolidated framework for advancing implementation science. Implement Sci. 2009;4(1):50. https://doi.org/10.1186/1748-5908-4-50
16. May C. A rational model for assessing and evaluating complex interventions in health care. BMC Health Serv Res. 2006;6:86. https://doi.org/10.1186/1472-6963-6-86
17. World Health Organization. A framework for implementing the set of recommendations on the marketing of foods and non-alcoholic beverages to children, 2012. https://apps.who.int/iris/bitstream/handle/10665/80148/9789241503242_eng.pdf?sequence=1. Accessed February 10, 2020
18. Meyers DC, Durlak JA, Wandersman A. The quality implementation framework: a synthesis of critical steps in the implementation process. Am J Community Psychol. 2012;50(3-4):462-480. https://doi.org/10.1007/s10464-012-9522-x
19. Sabatier PA. The advocacy coalition framework: revisions and relevance for Europe. J Eur Public Policy. 1998;5(1):98-130. https://doiorg/10.1080/13501768880000051
20. Domitrovich CE, Bradshaw CP, Poduska JM, et al. Maximizing the Implementation Quality of Evidence-Based Preventive Interventions in Schools: A Conceptual Framework. Adv Sch Ment Health Promot. 2008;1(3):6-28. https://doi.org/10.1080/1754730X.2008.9715730
21. World Health Organization. Regional Office for Europe. Steps to health: a European framework to promote physical activity for health. .WHO Regional Office for Europe, Copenhagen, 2007.
22. Chuang E, Ayala GX, Schmied E, Ganter C, Gittelsohn J, Davison KK. Evaluation Protocol To Assess an Integrated Framework for the Implementation of the Childhood Obesity Research Demonstration Project at the California (CA-CORD) and Massachusetts (MA-CORD) Sites. Child Obes. 2015;11(1):48-57. https://doi.org/10.1089/chi.2014.0049
23. May C,Finch T. Implementing, embedding, and integrating practices: An outline of Normalization Process Theory. Sociol. 2009;43(3), 535–554. https://doi.org/10.1177/0038038509103208

https://www.euro.who.int/__data/assets/pdf_file/0020/101684/E90191.pdf Accessed February 8, 2020

24. Hendriks A-M, Jansen MW, Gubbels JS, De Vries NK, Paulussen T, Kremers SP. Proposing a conceptual framework for integrated local public health policy, applied to childhood obesity - the behavior change ball. Implement Sci. 2013;8(1):46. https://doi.org/10.1186/1748-5908-8-46
25. Tumilowicz A, Ruel MT, Pelto G, et al. Implementation Science in Nutrition: Concepts and Frameworks for an Emerging Field of Science and Practice. Curr Dev Nutr. 2019;3(3):nzy080. https://doi.org/10.1093/cdn/nzy080
26. Kitson A, Harvey G, McCormack B. Enabling the implementation of evidence based practice: a conceptual framework. Qual Health Care QHC. 1998;7(3):149-158.
27. Weiner BJ, Lewis MA, Linnan LA. Using organization theory to understand the determinants of effective implementation of worksite health promotion programs. Health Educ Res. 2009;24(2):292-305. https://doi.org/10.1093/her/cyn019
28. Glasgow RE, Vogt TM, Boles SM. Evaluating the public health impact of health promotion interventions: the RE-AIM framework. Am J Public Health. 1999;89(9):1322-1327. https://doi.org/10.2105/ajph.89.9.1322
29. Maher D, Harries AD, Zachariah R, Enarson D. A global framework for action to improve the primary care response to chronic non-communicable diseases: a solution to a neglected problem. BMC Public Health. 2009;9(1):355. https://doi.org/10.1186/1471-2458-9-355
30. Campbell M, Fitzpatrick R, Haines A, et al. Framework for design and evaluation of complex interventions to improve health. BMJ. 2000;321(7262):694-696. https://doi.org/10.1136/bmj.321.7262.694
31. Poobalan AS, Aucott LS, Ahmed S, Smith WCS. Analysis of the UK recommendations on obesity based on a proposed implementation framework. BMC Public Health. 2010;10(1):17. https://doi.org/10.1186/1471-2458-10-17
32. Wandersman A, Duffy J, Flaspohler P, et al. Bridging the gap between prevention research and practice: the interactive systems framework for dissemination and implementation. Am J Community Psychol. 2008;41(3-4):171-181. https://doi.org/10.1007/s10464-008-9174-z
33. Oetzel J, Scott N, Hudson M, et al. Implementation framework for chronic disease intervention effectiveness in Māori and other indigenous communities. Glob Health. 2017;13(1):69. https://doi.org/10.1186/s12992-017-0295-8
34. Durlak JA, DuPre EP. Implementation matters: a review of research on the influence of implementation on program outcomes and the factors affecting implementation. Am J Community Psychol. 2008;41(3-4):327-350. https://doi.org/10.1007/s10464-008-9165-0
35. Orava T, Steve M, Rhona H. Support for healthy eating at schools according to the comprehensive school health framework: evaluation during the early years of the Ontario School Food and Beverage Policy implementation. Health Promot Chronic Dis Prev Can Res Policy Pract. 2017;37(9):303-312. https://doi.org/10.24095/hpcdp.37.9.05
36. Sharma SV, Upadhyaya M, Schober DJ, Byrd-Williams C. A conceptual framework for organizational readiness to implement nutrition and physical activity programs in early childhood education settings. Prev Chronic Dis. 2014;11:E190. https://doi.org/10.5888/pcd11.140166
37. Swinburn B, Egger G, Raza F. Dissecting obesogenic environments: the development and application of a framework for identifying and prioritizing environmental interventions for obesity. Prev Med. 1999;29(6 Pt 1):563-570. https://doi.org/10.1006/pmed.1999.0585
38. Atkins L, Francis J, Islam R, et al. A guide to using the Theoretical Domains Framework of behavior change to investigate implementation problems. Implement Sci. 2017;12(1):77. https://doi.org/10.1186/s13012-017-0605-9
